# Central Retinal Artery Occlusion in Acute Care: Current Practices and Emerging Therapies

**DOI:** 10.7759/cureus.92786

**Published:** 2025-09-20

**Authors:** Toufic Lakkis, Anas Mahmoud Awad Elshoura, Gabriel Andres Soria Behr, Mata Cardenas Eduardo Mauricio, Susana Sil-Zavaleta, Long Yin Cai, Manju Rai

**Affiliations:** 1 Internal Medicine, University of Balamand, Beirut, LBN; 2 Internal Medicine, Newcastle University, Newcastle upon Tyne, GBR; 3 Internal Medicine, University of Guayaquil, Guayaquil, ECU; 4 Internal Medicine, Autonomous University of the State of Hidalgo (UAEH), Hidalgo, MEX; 5 Dermatology, University La Salle Mexico City, Mexico City, MEX; 6 Dermatology, Hospital Ángeles del Pedregal, Mexico City, MEX; 7 Internal Medicine, Caribbean Medical University, Willemstad, CUW; 8 Biotechnology, Shri Venkateshwara University, Gajraula, IND

**Keywords:** acute retinal ischemia, artificial intelligence in diagnosis, central retinal artery occlusion (crao), emergency department (ed) management, nd:yag laser embolysis, neuro-ophthalmological emergency, ophthalmic stroke, optical coherence tomography (oct), pars plana vitrectomy (ppv), thrombolytic therapy

## Abstract

Central retinal artery occlusion (CRAO) represents a critical ophthalmological emergency characterized by sudden, painless monocular vision loss and requires immediate intervention within emergency department settings. This narrative review examines the current evidence regarding CRAO management, emphasizing its pathophysiology, risk factors, clinical presentation, diagnostic approaches, and therapeutic interventions. The condition's complex etiology encompasses both embolic and inflammatory processes, with significant associations to cardiovascular risk factors and systemic diseases. Despite its critical nature, CRAO management faces substantial challenges, including delayed patient presentation, limited therapeutic windows, and the absence of universally accepted treatment protocols. Current diagnostic approaches incorporate traditional fundoscopic examination alongside emerging technologies such as point-of-care ultrasound and optical coherence tomography. While conventional management strategies include ocular massage, intraocular pressure reduction, and systemic therapies, their efficacy remains limited by insufficient evidence from randomized controlled trials. Emerging therapeutic approaches, including targeted thrombolysis, hyperbaric oxygen therapy, and novel surgical interventions, show promise but require further investigation. The review emphasizes the importance of a multidisciplinary approach involving emergency medicine, ophthalmology, and neurology specialists to optimize patient outcomes. Future directions highlight the potential of artificial intelligence-assisted diagnostics, standardized "eye stroke" protocols, and ongoing research into novel therapeutic interventions. Success in addressing these challenges requires continued investigation through rigorous clinical trials, improved public awareness regarding CRAO symptoms, and enhanced emergency department protocols to facilitate rapid diagnosis and treatment initiation.

## Introduction and background

Central retinal artery occlusion (CRAO) represents a critical ophthalmological emergency characterized by obstruction of the central retinal artery (CRA), precipitating acute retinal ischemia and typically manifesting as sudden, painless unilateral vision loss [1]. The retina has a dual blood supply that is essential for its function and vulnerability to ischemia. The CRA (a branch of the ophthalmic artery that supplies the inner layers of the retina) nourishes the inner two-thirds of the retina, including the ganglion cells and inner plexiform layers that are vital for transmitting visual signals. In some individuals, a cilioretinal artery (an additional vessel arising from the posterior ciliary circulation that provides blood to the macula) supplements blood flow to the central retina and can serve as a collateral source of perfusion when the CRA is occluded. The outer retina, particularly the photoreceptor layer (cells responsible for detecting light), depends on the choroidal circulation supplied by the posterior ciliary arteries. Disruption of these vascular pathways compromises neuronal metabolism and leads to rapid neuronal degradation (damage and death of retinal nerve cells), which explains the sudden and often irreversible vision loss associated with CRAO.

The condition poses a substantial challenge in emergency department (ED) settings due to its time-sensitive management and limited therapeutic options. Epidemiological data indicate an annual incidence of 5.84 cases per 100,000 population, with a predilection for older individuals and those with cardiovascular risk factors such as hypertension, diabetes mellitus, and hyperlipidemia [2-3]. Beyond its ocular manifestations, CRAO also serves as a sentinel event signaling potential systemic disease, necessitating urgent diagnostic and management protocols [4-5]. Suboptimal awareness among healthcare providers has been linked to delays in recognition and intervention, thereby reducing the likelihood of preserving retinal function [6]. Experimental studies further highlight the retina’s limited ischemic tolerance, with neuronal degradation beginning approximately 240 minutes after occlusion [7].

This narrative review examines the importance of early recognition and intervention for CRAO in emergency medicine. It synthesizes current evidence on clinical presentation, risk stratification, and diagnostic markers, while also evaluating existing and emerging treatment options. By integrating ophthalmological and emergency care perspectives, the review aims to provide practical, evidence-based guidance to optimize patient outcomes and reduce the risk of secondary ischemic events.

## Review

Search methodology

A comprehensive literature search was conducted to identify studies relevant to CRAO, its risk factors, pathophysiology, diagnostic approaches, and management strategies. The search was performed in PubMed/MEDLINE, Embase, Scopus, and Web of Science databases, covering publications up to August 2025. Reference lists of included articles and related reviews were also screened to capture additional eligible studies.

The search strategy incorporated both Medical Subject Headings (MeSH) and free-text terms. Key MeSH terms included “central retinal artery occlusion,” “retinal artery occlusion,” “ischemic optic neuropathy,” “carotid artery diseases,” and “thrombolytic therapy.” Free-text keywords such as “CRAO,” “eye stroke,” “retinal ischemia,” “hyperbaric oxygen therapy,” “artificial intelligence imaging CRAO,” and “endovascular therapy CRAO” were also applied. Boolean operators (AND, OR) were used to combine terms, for example: (“central retinal artery occlusion” OR “CRAO” OR “eye stroke”) AND (“thrombolysis” OR “hyperbaric oxygen therapy” OR “diagnosis”).

Inclusion criteria comprised original research articles, clinical trials, observational studies, meta-analyses, systematic reviews, and narrative reviews that reported on CRAO epidemiology, risk factors, diagnostic modalities, management options, or future directions. Exclusion criteria were non-English publications, case reports or case series with fewer than five patients, editorials or commentaries lacking primary data, and studies focused on ocular vascular conditions unrelated to CRAO unless they provided mechanistic or comparative insights. The final selection prioritized studies with strong clinical relevance to CRAO diagnosis and management.

Risk factors

CRAO manifests through a multifactorial interplay of demographic, systemic, and physiological contributors. Advanced age, particularly in individuals over 50, represents a significant predisposition, with males experiencing slightly higher incidence rates [8].

Cardiovascular Risk Factors

Hypertension, diabetes mellitus, hyperlipidemia, obesity, and chronic smoking are critical contributors to CRAO [9]. Atherosclerosis serves as the primary underlying mechanism, with associated cardiovascular diseases such as coronary artery disease and cerebrovascular conditions substantially increasing susceptibility [10]. Structural abnormalities, including carotid artery stenosis, cardiac valve disorders, patent foramen ovale, and atrial fibrillation, can generate embolic material capable of obstructing retinal circulation [1].

Inflammatory Risk Factors

Inflammatory disorders also compromise vascular integrity and predispose to occlusive events. Conditions such as giant cell arteritis, systemic lupus erythematosus, and polyarteritis nodosa have been strongly associated with CRAO development [11].

Hematological and Thrombotic Risk Factors

Hypercoagulable states, including antiphospholipid syndrome and genetic mutations such as factor V Leiden, significantly augment occlusion probability [12]. Hematological disorders, such as sickle cell disease and polycythemia vera, create prothrombotic environments conducive to retinal arterial blockage [13]. Iatrogenic risks, including surgical interventions and intravenous drug use, also contribute to thrombotic risk [14].

Lifestyle and Other Risk Factors

Lifestyle factors remain important in CRAO risk stratification. Physical inactivity, chronic stress, poor dietary habits, and excessive alcohol consumption contribute to systemic inflammatory and thrombotic processes [15]. Genetic predispositions, including familial cardiovascular disease history and inherited thrombophilia, provide additional contextual risk [16]. Less common but notable associations include migraine with aura, hyperhomocysteinemia, sleep apnea, and oral contraceptive use [17].

In addition to identifying established risk factors, preventive strategies aimed at early detection and risk modification are critical. Carotid artery disease is a significant contributor to embolic events leading to CRAO, and targeted screening in high-risk populations can help identify asymptomatic carotid stenosis. Current recommendations by the American Heart Association (AHA) emphasize risk-based assessment, advising against general population screening but supporting carotid ultrasound in individuals with multiple vascular risk factors, history of transient ischemic attack, or other clinical indicators of cerebrovascular disease [18]. Incorporating such preventive strategies into clinical practice may allow earlier intervention with medical therapy, lifestyle modification, or, in selected cases, revascularization procedures, thereby reducing the risk of retinal and cerebral ischemic events.

Pathophysiology

**Figure 1:**
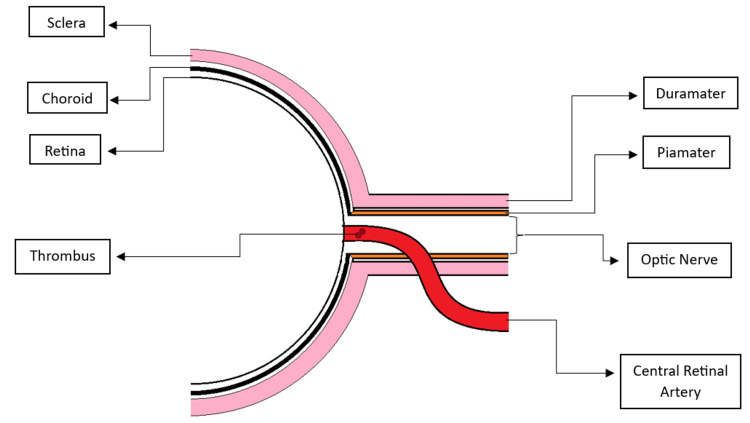
Schematic representation of central retinal artery occlusion (CRAO) illustrating thrombus formation within the central retinal artery, leading to retinal ischemia. Figure created by Susana Sil-Zavaleta

The retina relies on two distinct circulatory systems: the CRA, which supplies the inner two-thirds, and the choroidal circulation, which nourishes the outer third, including photoreceptors. The CRA arises from the internal carotid via the ophthalmic artery, enters the eye through the optic nerve, and branches into arterioles serving the inner retina. Because these arterioles lack collateral circulation and have narrow lumina, they are highly susceptible to obstruction by even small emboli from carotid or ophthalmic plaques [19]. CRAO etiopathogenesis encompasses two major mechanisms (Table 1). Non-arteritic CRAO, the more common form, usually results from emboli originating in the internal carotid artery [20] or from local thrombus formation in hypercoagulable states [21] (Figure 1). Arteritic CRAO arises from inflammatory vasculitides such as giant cell arteritis, polyarteritis nodosa, or systemic lupus erythematosus, where vessel inflammation restricts perfusion [22]. A rare manifestation, termed “retinal migraine,” arises from vasospasm of the CRA, leading to transient episodes of monocular vision loss that typically resolve spontaneously. In most cases, visual symptoms are short-lived; however, in a minority of patients, prolonged vasospasm can compromise retinal perfusion and result in permanent visual deficits [23-24].

**Table 1:** Central retinal artery occlusion (CRAO) types and visual prognosis.

Type	Percentage	Description	% Vision regain
Nonarteritic CRAO [9-10]	66%	Caused by a permanent occlusion of the central retinal artery due to an embolus at the narrowest part of its course at its entry into the optic sheath.	<20%
Nonarteritic CRAO with cilioretinal sparing [10]	13%	Involves sparing of the cilioretinal artery despite CRAO.	Over 80% achieve 20/50 or better vision
Transient non-arteritic CRAO [10]	16%	A temporary blockage of the central retinal artery lasting minutes to hours, caused by factors such as a migrating embolus, increased intraocular pressure, or a severe drop in perfusion.	82% improve within the first week
Arteritic CRAO [11]	5%	Caused by occlusion of the common trunk of the central retinal artery and posterior ciliary artery, typically due to giant cell arteritis. Usually present with risk of symptomatic ischemic stroke after two weeks of CRAO.	Poor visual recovery

Downstream consequences of occlusion reflect both acute and chronic ischemia. Early cellular death triggers retinal edema, intensifying hypoxia and leading to rapid ganglion cell and nerve fiber layer loss [19]. Over time, unresolved ischemia results in inner retinal thinning and optic atrophy, producing irreversible vision deterioration [25]. These mechanisms underscore the urgency of early recognition and intervention to minimize ischemic duration and optimize patient outcomes.

Clinical presentation

RAO arises from obstruction within the ophthalmic artery or its branches, most commonly the CRA, and typically presents as acute, painless monocular vision loss with an afferent pupillary defect [26-27]. If a cilioretinal branch supplies the fovea, vision loss may be partial [26]. Diagnostic delays are frequent due to the painless nature of symptoms and limited awareness among healthcare providers [28]. Epidemiological data from the American Academy of Ophthalmology IRIS registry, encompassing over one million vascular occlusions, reported 296,887 RAO cases, with a predilection for older males and right-sided involvement, possibly reflecting anatomical connections between the ophthalmic artery and the brachiocephalic trunk [29].

A validated questionnaire developed by Casagrande et al. identified abrupt vision loss, dark shadows in the visual field, and onset within seconds as highly specific for CRAO [30]. Fundoscopic signs may be absent in the initial hours, though patients typically present beyond 4.5 hours, when retinal pallor, macular cherry spot, delayed vessel filling, and early capillary non-perfusion become evident on fluorescein angiography [26,31].

Differential diagnoses include amaurosis fugax (AF) and retinal vein occlusion. CRAO is distinguished from AF by vision loss lasting longer than five minutes, though comorbidity profiles are often comparable [28]. Combined artery and vein occlusion may occur, presenting with more extensive retinal involvement, dilated veins, optic disc edema, and oscillating arteriovenous filling [31]. Iatrogenic filler embolism to the ophthalmic artery is an important cause, presenting as painful monocular vision loss post-injection [32]. Chronic glaucoma, with optic disc changes, field defects, and elevated intraocular pressure (IOP), may also mimic CRAO [33].

Diagnosis in the emergency department

Patients with CRAO typically present with acute, painless monocular vision loss, often accompanied by an afferent pupillary defect and impaired color vision. Extraocular movements, IOPs, and anterior chamber findings are usually normal [34-36]. Atherosclerotic risk factors are common, with hyperlipidemia (36%), hypertension (27%), and diabetes (12%) most frequently reported; however, in younger patients or those without vascular risks, alternative causes such as vasculitis, sickle cell disease, hypercoagulable states, or drug exposure should be considered [37].

Fundoscopic changes evolve over time. Early findings include cherry-red spot (90%), retinal pallor (39%), posterior pole opacity (58%), arterial attenuation (32%), optic disc edema (22%), and cattle trucking sign (19%). Chronic changes may show optic atrophy (91%), persistent arterial attenuation (58%), and cilioretinal collaterals. Examination of the fellow eye is important, as CRAO may be associated with central scotoma (19%) or temporal island defects (59%) [7,38].

Adjunctive imaging enhances diagnostic accuracy. Point-of-care ultrasound (POCUS) can rapidly identify the retrobulbar spot sign in embolic CRAO [39-40]. Optical coherence tomography (OCT) and OCT angiography (OCT-A) demonstrate inner retinal thickening in acute cases, with variable findings such as intraretinal fluid or neurosensory detachment in severe disease [41-42]. Fluorescein angiography reveals delayed filling, reduced arterial caliber, and cattle trucking of branch arteries.

Comprehensive workup should include vascular risk evaluation, neuroimaging, and targeted labs. Patients under 50 years without vascular risk factors warrant additional testing for hypercoagulable or hematologic disorders [36]. Recent advances, including the integration of OCT in stroke centers and EDs, allow rapid imaging and remote specialist interpretation, improving early detection and timely intervention in up to 42% of cases [43]. Table 2 summarizes key diagnostic modalities for CRAO in emergency settings.

**Table 2:** Diagnosis of central retinal artery occlusion (CRAO) in the emergency department.

Diagnostic modality	Findings	Clinical utility
Clinical examination [36]	Acute, painless monocular vision loss, afferent pupillary defect, color vision impairment.	Initial assessment; helps differentiate from other vision loss causes.
Fundoscopic examination [37]	Early: Cherry-red spot (90%), retinal opacity (58%), arterial attenuation (32%), optic disc edema (22%), cattle trucking (19%). Late: Optic atrophy (91%), arterial attenuation (58%), cilioretinal collaterals.	Time-dependent changes confirm diagnosis and disease stage.
Point-of-care ultrasound (POCUS) [38-39]	Retrobulbar spot sign in thromboembolic CRAO.	Rapid, non-invasive bedside diagnosis; differentiates CRAO from other causes.
Optical coherence tomography (OCT) [40-41]	Acute: Inner retinal layer thickening. Mild: Middle retinal layer opacification. Severe: Intraretinal fluid, neurosensory detachment, posterior vitreous opacities.	Provides objective assessment within the thrombolysis window.
Fluorescein angiography [35]	Delayed vessel filling, reduced arterial caliber, cattle trucking of blood column.	Identifies perfusion defects and vascular occlusion patterns.
Laboratory testing [35]	Hyperlipidemia (36%), hypertension (27%), diabetes (12%), hypercoagulable states, vasculitis markers.	Evaluates underlying vascular and systemic risk factors.
Neuroimaging (CT/MRI, carotid Doppler) [35]	Identifies atherosclerotic plaques, emboli, and stroke risk.	Essential for systemic stroke workup and secondary prevention.
Advanced diagnostic approaches [42]	OCT integration in emergency/stroke centers - 42% improvement in early detection and treatment.	Enhances rapid assessment and remote interpretation for CRAO cases.

Emergency management strategies

Immediate Interventions

Ocular massage is an easily accessible and rapid intervention that may help dislodge the embolus and restore retinal perfusion. It involves applying intermittent, moderate-pressure digital massage to the closed eyelid for 10-15 seconds, followed by brief releases, to enhance retinal circulation by inducing fluctuations in IOP and promoting embolus movement into a more distal artery [26]. However, its efficacy remains uncertain, with limited success reported in clinical studies [44].

Lowering IOP is another crucial early intervention aimed at increasing retinal perfusion pressure. Systemic carbonic anhydrase inhibitors such as acetazolamide (500 mg IV or PO) and topical beta-blockers (e.g., timolol 0.5%) are frequently administered to reduce aqueous humor production and facilitate retinal blood flow [45]. Additionally, intravenous mannitol and oral glycerol have been employed in severe cases, though evidence supporting their use is limited [46].

Anterior chamber paracentesis involves removing a small volume of aqueous humor (0.1-0.2 mL) via a fine-gauge needle to rapidly decrease IOP and potentially improve retinal perfusion [47]. While considered a low-risk procedure, it requires expertise and carries a minimal risk of infection or anterior chamber collapse. Despite its theoretical benefits, studies suggest that it provides only modest improvement in visual outcomes [48].

Hyperbaric oxygen therapy (HBOT) enhances oxygen delivery to ischemic retinal tissue and may improve visual prognosis if administered within 24 hours of symptom onset [49]. It involves breathing 100% oxygen at high pressure, increasing oxygen diffusion to the retina via the choroidal circulation [50]. Evidence from case series suggests HBOT may be beneficial when used promptly, though randomized trials are lacking [51]. A recent literature review synthesizing therapeutic strategies for retinal artery occlusion further supports the role of thrombolysis and HBOT as promising interventions [52]. This review incorporates updated meta-analyses, reinforcing the importance of timely reperfusion strategies and providing practical perspectives on integrating pharmacological and adjunctive therapies into emergency protocols.

Systemic Therapies

The rationale for using antiplatelet and anticoagulation therapy in CRAO is based on its embolic etiology, often linked to atherosclerosis or carotid artery disease. Aspirin, clopidogrel, and other antiplatelet agents are commonly prescribed to reduce the risk of recurrent vascular events [53]. However, randomized controlled trials (RCTs) directly evaluating antiplatelet efficacy in CRAO are lacking. Retrospective studies suggest that early aspirin administration may lower the risk of subsequent strokes, but visual recovery remains poor [26].

Anticoagulation, particularly with heparin or warfarin, has been explored in cases associated with cardioembolic sources, such as atrial fibrillation. The European Assessment Group for Lysis in the Eye (EAGLE) study, a prospective trial, assessed heparin versus conservative management in acute CRAO but found no significant benefit in visual outcomes [54]. A retrospective analysis of CRAO patients with atrial fibrillation suggested that systemic anticoagulation might reduce the incidence of recurrent embolic events but did not substantially improve visual prognosis [55].

Intravenous thrombolysis with tissue plasminogen activator (tPA) has been investigated as an emergent intervention for CRAO. An observational cohort demonstrated a trend toward improved visual acuity with tPA but raised safety concerns due to a risk of intracranial hemorrhage [56]. Another large retrospective study indicated that tPA use within 4.5 hours of symptom onset was associated with better visual recovery, though definitive RCTs are lacking [57].

More recent trials, such as THrombolysis with alteplase in patients with acutE central retInal Artery occlusion (THEIA) (NCT03197194), are currently investigating whether tPA administration improves outcomes when administered within six hours of CRAO onset [58]. Given mixed evidence, current guidelines remain cautious, emphasizing individualized risk-benefit assessment.

Role of Emerging Therapies

In addition to the widely used treatments, several emerging therapies aim to enhance management strategies for CRAO by minimizing damage and preserving ocular function. One such approach involves thrombolytic agents, which dissolve thrombi or emboli. Wilkins et al. demonstrated the successful restoration of blood flow to the left CRA in a patient with Barlow’s disease, despite an occlusion persisting for over 10 hours [59].

Similarly, Margolin et al. reported a case where intravascular intervention was performed within two to three hours of symptom onset. Following the administration of a thrombolytic agent to a partially occluded artery, the patient experienced near-immediate visual recovery [60]. Mathew et al. also documented the successful use of alteplase to resolve occlusions in multiple cranial arteries, including the CRA. These occlusions were attributed to severe aortic valve calcification and atherosclerosis of the aortic arch. Post-treatment, the patient’s vision was fully restored to 20/20 [61].

Experimental models have further explored potential treatments; Agarwal et al. conducted a study on six adult rhesus monkeys (12 eyes) with induced CRAO, demonstrating improved circulation following tPA administration [62]. Efforts to mitigate ischemic damage post-CRAO have also focused on retinal restoration. Gao et al. used a rat model to investigate the effects of mesenchymal stem cells and extracellular vesicles (MSC-EV) administered into the vitreous humor 24 hours after retinal ischemia [63]. Their findings suggest that MSC-EV may promote functional recovery, reduce neuroinflammation and apoptosis, and minimize retinal damage and cellular loss.

Additionally, natural compounds have shown promise in neuroprotection. Kim et al. investigated the effects of KIOM-2015E, an extract from Acer palmatum thumb leaves, and found that it significantly reduced retinal ganglion cell loss and nerve fiber degeneration, among other protective effects on retinal cells [64].

Challenges in the emergency department

**Figure 2:**
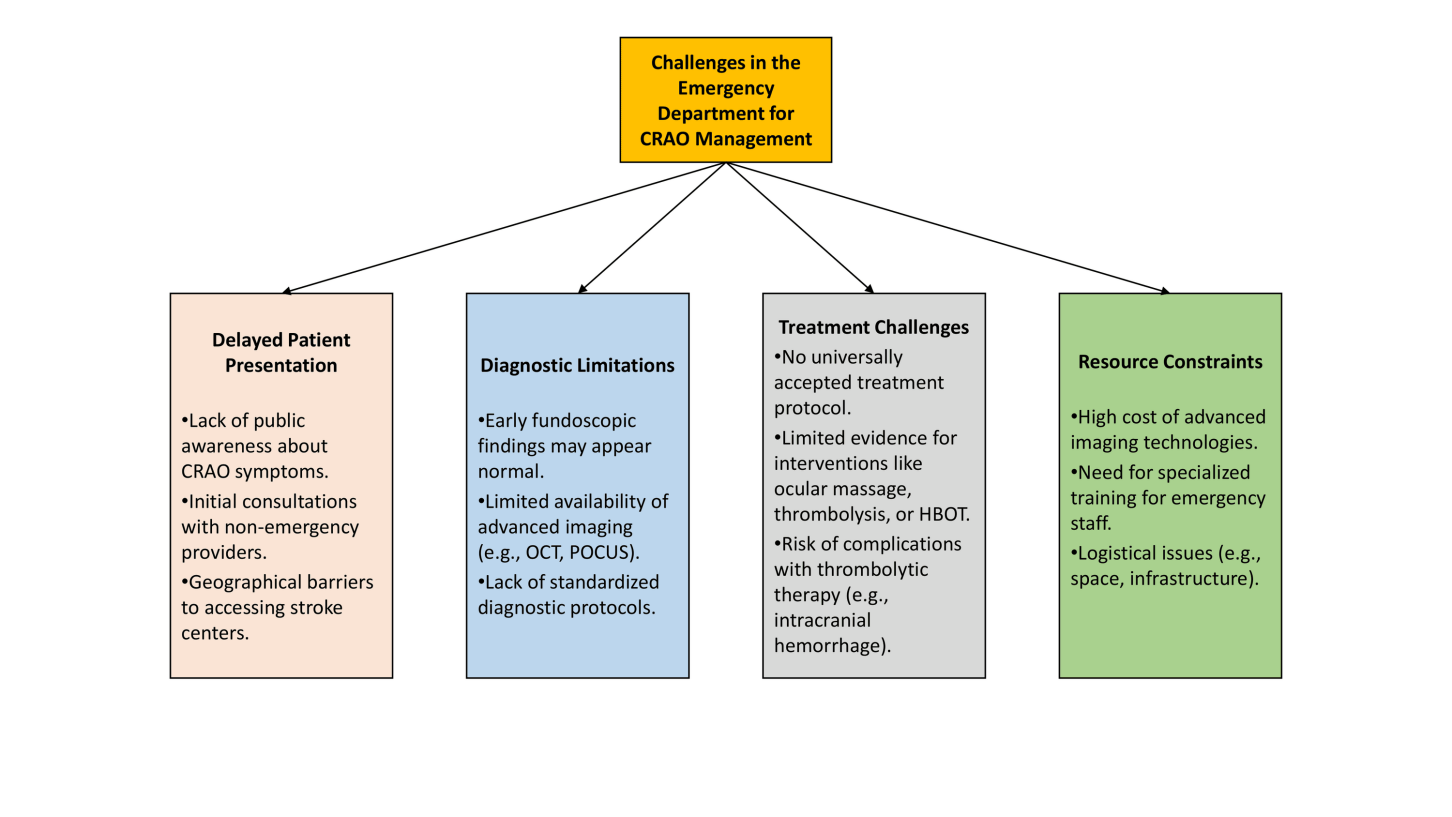
Challenges in central retinal artery occlusion (CRAO) management in the emergency department. Figure created by Anas Mahmoud Awad Elshoura

The management of CRAO in the ED is fraught with challenges due to the condition’s time-sensitive nature and the limited availability of effective treatment options (Figure 2). 

Delayed Patient Presentation

A major obstacle in CRAO management is delayed patient presentation. Contributing factors include lack of public awareness regarding the urgency of sudden vision loss, initial consultations with non-emergency providers delaying referral, and geographical barriers limiting access to tertiary care centers or specialized stroke units [65-66].

Diagnostic Limitations

Diagnosing CRAO in the hyper-acute phase is challenging, as fundoscopic examination may initially appear normal. Advanced imaging tools such as retinal OCT and non-mydriatic ocular fundus photography (NMFP) can enable early recognition, yet these technologies are not consistently available across ED settings [67]. Their integration into emergency workflows, through "eye stroke" protocols, could improve timely diagnosis and intervention [68].

Barriers to Advanced Imaging

Despite their utility, advanced imaging technologies face barriers including high costs, need for specialized training, workflow integration difficulties, absence of standardized protocols, and infrastructure constraints. Overcoming these challenges requires awareness, funding, and clear guidelines for ED implementation [67-68].

Thrombolysis Challenges

The role of thrombolytic therapy in CRAO remains controversial. While some evidence suggests potential visual benefit when administered within a narrow therapeutic window, data remain inconclusive, and risks such as symptomatic intracranial or extracranial hemorrhage are concerning [69]. Intra-arterial tissue plasminogen activator (IA-tPA) has shown promise in select cases, but its efficacy and safety have yet to be confirmed in high-quality RCTs [27,70-71]. Recent consensus statements, including the 2025 AHA update, suggest improved visual outcomes when intravenous thrombolysis is given within 4.5 hours and intra-arterial therapy within six hours of onset, but real-world implementation is hampered by late presentation and limited stroke-center readiness [18]. Future research is essential to clarify patient selection criteria, refine treatment windows, and balance risks with potential benefits.

Therapeutic Uncertainty

The absence of consensus on effective treatment strategies further complicates CRAO management. Traditional interventions such as ocular massage, anterior chamber paracentesis, and HBOT lack proven benefit [69,72]. Thrombolytic therapy remains controversial, with limited supporting evidence and risks including intracranial or extracranial hemorrhage [69]. Careful patient selection and risk-benefit assessment are essential when considering such therapies.

Underlying Systemic Risk Factors

Most CRAO cases are embolic in origin, with plaques from carotid arteries, heart valves, or the aortic arch serving as common sources [69]. These Hollenhorst plaques obstruct the CRA, producing acute vision loss. Real-world experiences highlight the benefits of standardized emergency protocols; for example, a 2024 AAO EyeNet report described improved imaging, vascular risk evaluation, and selective vision preservation with stroke-like response systems [73].

Multidisciplinary Care Needs

CRAO’s systemic implications necessitate a multidisciplinary approach. Internists play a key role, as CRAO may be the first manifestation of systemic disease. AAO EyeNet reported CRAO as an initial sign of systemic lupus erythematosus [74], while Brar et al. described bilateral CRAO with tongue necrosis in giant cell arteritis [75]. Infective sources also contribute; Serras-Pereira et al. reported CRAO caused by embolization from *Streptococcus gallolyticus* endocarditis [76].

Neurologists assist in differentiating non-arteritic CRAO from neurological conditions, with transorbital ultrasound and adapted stroke protocols showing promise [5,77-78]. Cosmetic specialists must recognize the growing association between CRAO and ophthalmic artery occlusion from facial filler injections [79]. Ophthalmologists remain central to diagnosis and management, with emerging biomarkers such as ApoA1, high-density lipoprotein cholesterol (HDL-C), and metabolic compounds (PC (P-18:0/20:4), PC (P-18:0/22:6), and octanylcarnitine) offering potential for early risk prediction [80].

This multidisciplinary collaboration underscores the complexity of CRAO management and the importance of coordinated expertise to optimize patient outcomes.

Future directions

Future advancements in CRAO management should focus on addressing key challenges, including delayed patient presentation, diagnostic limitations, and the absence of standardized treatment protocols.

Near-Term Advances

The integration of advanced diagnostic technologies, such as OCT and NMFP, has the potential to facilitate rapid and accurate diagnosis of CRAO in the ED. A remote consultation model, where OCT images are interpreted by specialists off-site, has demonstrated improved diagnostic accuracy and reduced time to treatment, thereby mitigating the challenge posed by the limited availability of ophthalmologists in emergency settings [67]. Further enhancement of diagnostic capabilities may be achieved through artificial intelligence (AI)-assisted imaging analysis. A study by Lema et al. demonstrated that machine learning algorithms (MLAs) trained on OCT images were able to detect changes in retinal layer thickness and opacity with high discriminative power, enabling the rapid differentiation of CRAO from normal retinal findings [43]. The integration of AI into routine clinical workflows could significantly improve early recognition and expedite management.

Public awareness campaigns emphasizing the urgency of sudden vision loss as a potential stroke warning sign are critical for reducing prehospital delays. Encouraging individuals to seek immediate medical attention may increase the likelihood of presenting within the therapeutic window for potential interventions, thereby improving patient outcomes [66]. Standardizing "Eye Stroke" protocols in EDs is another essential step in optimizing CRAO management. These protocols should emphasize rapid identification, immediate diagnostic imaging, and timely initiation of treatment, which may enhance visual outcomes and streamline care pathways [67,81].

The use of thrombolytic therapy, particularly IA-tPA, remains controversial. While some studies suggest that thrombolysis may offer benefits if administered within a specific time frame, concerns regarding potential risks persist. Future research should focus on conducting high-quality randomized clinical trials to determine the efficacy and safety of IA-tPA for CRAO treatment [27,70-71].

Long-Term Experimental Strategies

Emerging therapeutic interventions under investigation include Nd:YAG laser embolysis and pars plana vitrectomy (PPV). Nd:YAG laser embolysis utilizes infrared laser energy to fragment emboli, with studies reporting visual improvement in 87% of patients undergoing the procedure for retinal artery occlusions, including CRAO [82]. PPV, when performed under low infusion pressure, facilitates IOP reduction, increasing perfusion pressure within the CRA, thereby aiding in embolus dislodgement. Additionally, a novel approach combining PPV with endovascular surgery involves inserting a microneedle into the retinal artery at the optic disc post-PPV, followed by the administration of 0.1 to 0.2 mL of tPA. Unlike conventional intra-arterial thrombolysis, this technique considers the simultaneous presence of white arteries and veins as an indicator of successful treatment [83].

Ongoing research into novel pharmacological agents and therapeutic targets for retinal ischemia may further expand treatment options. Potential strategies include restoring retinal perfusion, enhancing oxygen delivery, and minimizing ischemic injury [84-86]. Given the current lack of established therapies for acute CRAO, future investigations should evaluate the impact of CRA thrombolysis on visual outcomes, determine the optimal therapeutic window for IA-tPA administration, and compare its efficacy and safety against emerging interventions such as Nd:YAG laser embolysis and PPV. Additionally, studies should assess potential complications, including ocular neovascularization and neovascular glaucoma, to ensure safer and more effective treatment approaches.

## Conclusions

This comprehensive review highlights the urgent need for prompt recognition and management of CRAO within ED settings. Despite advances in awareness, significant challenges persist, including delayed patient presentation, limited diagnostic availability in the hyperacute phase, and the absence of universally accepted treatment protocols. These barriers continue to restrict the effectiveness of existing interventions. However, recent developments in advanced imaging modalities, AI-assisted diagnostics, and the implementation of standardized “Eye Stroke” pathways represent important steps toward earlier recognition and timely intervention. Alongside these advances, ongoing research into thrombolysis, HBOT, and emerging techniques such as laser embolysis provides new opportunities for improving visual prognosis and reducing the risk of subsequent ischemic events.

Looking ahead, the priorities in CRAO management must include strengthening public education campaigns to emphasize the urgency of sudden vision loss, integrating rapid diagnostic protocols into emergency workflows, and pursuing rigorous clinical trials to clarify the efficacy and safety of potential therapies. Achieving these goals will require robust multidisciplinary collaboration involving emergency physicians, ophthalmologists, neurologists, internists, and allied specialists, as well as institutional commitment to developing and implementing standardized care pathways. By addressing current challenges and harnessing advances in both diagnostics and therapeutics, the healthcare community can move closer to optimizing outcomes for patients affected by this devastating yet time-sensitive condition.
